# Can Stoss Therapy Be Used in Children with Vitamin D Deficiency or Insufficiency without Rickets?

**DOI:** 10.4274/jcrpe.3842

**Published:** 2017-06-01

**Authors:** Cemil Koçyiğit, Gönül Çatlı, Gülberat İnce, Elif Büşra Özkan, Bumin Nuri Dündar

**Affiliations:** 1 İzmir Katip Çelebi University Faculty of Medicine, Department of Pediatric Endocrinology, İzmir, Turkey; 2 Tepecik Training and Research Hospital, Clinic of Pediatrics, İzmir, Turkey; 3 İzmir Katip Çelebi University Faculty of Medicine, İzmir, Turkey

**Keywords:** Vitamin D deficiency, rickets, stoss therapy

## Abstract

**Objective:**

Stoss vitamin D treatment has been recommended for its non-skeletal benefits in adults, but there is a lack of data on the optimal dose of vitamin D stoss therapy in children with vitamin D deficiency/insufficiency without rickets. This study aimed to compare efficiency/side effects of two different stoss therapy regimens (10 000 IU/kg and 300 000 IU vitamin D3) administered in children with vitamin D deficiency/insufficiency without rickets.

**Methods:**

Sixty-four children who had vitamin D deficiency/insufficiency were studied. A serum 25-hydroxyvitamin-D (25-OH-D) level of 15-20 ng/mL was considered as vitamin D insufficient and <15 ng/mL was considered as vitamin D deficient. The patients were divided into two groups according to the stoss therapy doses they received. Serum calcium, phosphate, alkaline phosphatase, 25-OH-D, parathyroid hormone levels, and spot urine calcium/creatinine ratios before/after treatment were recorded. Wrist radiography and renal ultrasonography were performed.

**Results:**

The mean age of the subjects was 10.6±4.4 years. Thirty-two children were treated with a single vitamin D3 dose of 10 000 IU/kg and 32 patients received 300 000 IU. No difference was found in 25-OH-D levels between the two groups at presentation. The mean level of 25-OH-D was higher in the 10 000 IU/kg group at the second week of therapy. There was no difference between the groups at post-treatment weeks 4 and 12. The 25-OH-D was found to be below optimal levels (≥30 ng/mL) in 66.5% and <20 ng/mL in 21.8% of patients at the third month in both groups. None developed hypercalcemia and/or hypercalciuria. Nephrolithiasis was not detected in any patient.

**Conclusion:**

This study showed that both doses of stoss therapy used in the treatment of vitamin D insufficiency/deficiency are effective and safe. However, an optimal level of 25-OH-D cannot be maintained for more than three months.

## What is already known on this topic?

Due to the proven benefits of vitamin D outside the skeletal system, physicians use stoss therapy in their adult patients with vitamin D deficiency/insufficiency.

## What this study adds?

This study shows that stoss therapy regimens (10 000 IU/kg and 300 000 IU vitamin D_3_) can be used safely in children with vitamin D deficiency/insufficiency without rickets.

## INTRODUCTION

Vitamin D is essential for mineralization of bones, for calcium and phosphate homeostasis, and neuromuscular conduction (1). Low vitamin D levels lead not only to rickets in children and osteomalacia in adults but also to muscle weakness and predisposition to respiratory infections, hyperparathyroidism, inability to acquire peak bone mass, and increased risk of fracture (2). Many clinicians now measure vitamin D levels as part of routine laboratory workup and recommend vitamin D supplements often at high doses to their patients for the possible prevention of cancer, cardiovascular disease, diabetes, autoimmune disorders, and other conditions since observational studies support the speculation that there is a relationship between low vitamin D levels and an increased risk for these situations (3). Stoss treatment, namely, the administration of vitamin D in a high dose (300 000-600 000 IU) has also been suggested in adults due to the non-skeletal effects of vitamin D, but no sufficient data are present for children with vitamin D deficiency/insufficiency without rickets. Thus, an optimal treatment dose of vitamin D is not well known in patients with vitamin D deficiency or insufficiency who do not show marked signs of rickets. Despite the worldwide programs of vitamin D supplementation as a public health campaign, a considerable number of children are still at high risk because of the poor adherence of the parents to supplementation regimens. Because the signs and symptoms of vitamin D deficiency without rickets are insidious or nonspecific, it often goes unrecognized and untreated (4).

In this retrospective study, the efficacy and side effects of two different doses of stoss therapy (10 000 IU/kg and 300 000 IU, oral, single-dose vitamin D_3_) in vitamin D deficient or insufficient children without marked signs of rickets were compared.

## METHODS

The children and adolescents who had been referred to the pediatric endocrinology department due to vitamin D deficiency or insufficiency between January 2014 and January 2015 and who received stoss therapy in two different doses (10 000 IU/kg and 300 000 IU, oral, single-dose vitamin D_3_) were studied retrospectively (5). Patients with chronic diseases such as malabsorption, liver disease, renal disease, gastrointestinal, hematologic and rheumatologic diseases, and those using drugs which may influence vitamin D metabolism were excluded from the study. Age, gender, anthropometric measurements, season of admission, and complaints at presentation of the patients were recorded. A serum level of 25-hydroxyvitamin D (25-OH-D) between 15-20 ng/mL was accepted as vitamin D insufficiency, <15 ng/mL as vitamin D deficiency, and <5 ng/mL as severe vitamin D deficiency. The serum levels of calcium (Ca), phosphate (P), alkaline phosphatase (ALP), 25-OH-D, parathyroid hormone (PTH), spot urine calcium/creatinine (UCa/UCr) ratio before and after treatment (weeks 2, 4 and 12), and renal ultrasonography (USG) outcomes in the three groups were compared. Clinical complaints that brought patients to the clinics were recorded and information about whether these complaints continued two weeks after vitamin D therapy was obtained from the patient records. Laboratory reference values based on age groups were used for defining hypocalcemia, hypophosphatemia, elevated ALP, and hyperparathyroidism. UCa/UCr >0.2 in spot urine was considered as hypercalciuria. Serum Ca, P, and ALP levels were measured using the calorimetric method. Serum PTH and 25-OH-D levels were measured by electrochemiluminescense enzyme immunoassay method (ADVIA Centaur, USADPC Co., USA).

### Statistical Analysis

Statistical analysis was performed using SPPS 21.0 (SPSS Inc., Chicago, IL, USA) software. All data were given as mean ± standard deviation scores (SDS). Homogeneity of the data was assessed using the Kolmogorov-Smirnov test. Differences in the mean level of vitamin D between the two treatment groups (10 000 IU/kg and 300 000 IU) and between patients with or without symptoms were tested using the student’s t-test for data with normal distribution and the Mann-Whitney U test for data without normal distribution. The chi-square test was used to compare the number of patients with complaints before and after treatment. The results were expressed with a 95% confidence interval and a p-value of less than 0.05 was considered statistically significant.

## RESULTS

**Figure 1:**
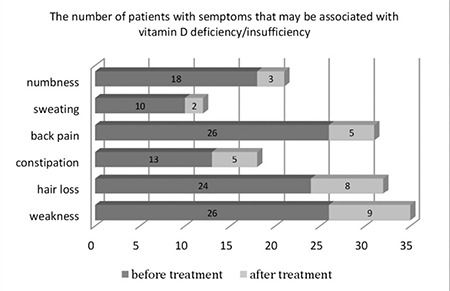
Numbers of patients with symptoms that may be associated with vitamin D status before and two weeks after treatment

**Figure 2:**
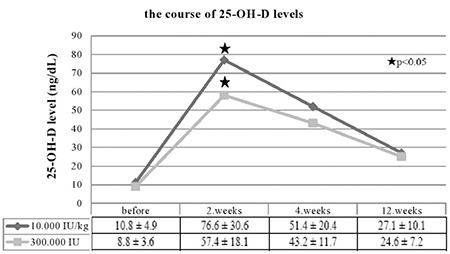
The course of 25-hydroxyvitamin-D levels over time in patients on the two different high-dose treatment protocols

Sixty-four patients of a mean age of 10.6±4.4 years were included in the study. Age, gender, anthropometric measurements, season of admission, and laboratory data of the patients are shown in Table 1. Of the patients, 32 were treated with 10 000 IU/kg (max. 600 000 IU) and the remaining 32 patients received a single 300 000 IU dose oral vitamin D_3_. Severe vitamin D deficiency was determined in 13, vitamin D deficiency in 42, and vitamin D insufficiency in 9 patients. Ca and P levels were in normal ranges in all patients, while the level of ALP was high in 12 and PTH was high in 8 patients. The mean 25-OH-D levels of the groups (10 000 IU/kg and 300 000 IU) were not significantly different before treatment (10.8±4.9 and 8.8±3.6 ng/mL, respectively, p>0.05). Of the patients, 26.6% (n=17) were asymptomatic. The most common symptoms at presentation were weakness (40.6%), lower back pain (40.6%), hair loss (37.5%), numbness in hands and feet (28.1%), constipation (20.3%), excessive sweating (15.6%), and frequent respiratory tract infection (12.5%). None of the patients had a history of a clinically significant fracture. More than one complaint was observed in 56% of patients. When the patients were re-evaluated two weeks after treatment, the number of patients with symptoms was found to be significantly reduced (p<0.05) (Figure 1). The mean level of 25-OH-D was significantly higher in the 10 000 IU/kg group at the second week after treatment (76.6±30.6 vs. 57.4±18.1 ng/mL, p<0.05), but there were no statistically significant differences between the groups in the levels of vitamin D at the 4^th^ and 12^th^ weeks after treatment (p>0.05) (Figure 2). The level of 25-OH-D was reduced below optimal levels (≥30 ng/mL) in 66.5% and below 20 ng/mL in 21.8% of the patients at 12 weeks post-treatment. None of the patients in either group developed hypercalcemia, hypercalciuria, or vitamin D intoxication. Nephrolithiasis was not detected in any of the patients at the third month of the treatment.

## DISCUSSION

he blood level of 25-OH-D defined as vitamin D deficiency remains somewhat controversial. As determined by the measurement of serum concentrations of calcidiol (25-OH-D), vitamin D deficiency is accepted to be present when values are below 15 ng/mL. In children, calcidiol concentrations between 15 and 20 ng/mL indicate vitamin D insufficiency, whereas those >20 ng/mL are adequate or sufficient (6,7). However, these guidelines, based on the recommendations of the Institute of Medicine report, are not accepted by all authorities and remain the subject of ongoing investigations. Alternate guidelines state that a normal 25-OH-D concentration be defined as greater than 30 ng/mL with values of 20 to 30 ng/mL used to define insufficiency and values of less than 20 ng/mL considered as vitamin D deficiency, especially for adults (8). Vitamin D deficiency in the infant may be managed by administration of vitamin D 1000 to 5000 IU/day. For the child who is one year of age or older, or an adolescent, vitamin-deficient rickets may be treated with 5000 to 10000 IU/day of vitamin D (9). Alternative therapeutic regimens for treatment of rickets include 50 000 IU orally weekly for 8 weeks, the administration of a single oral (or intramuscular) dose of 150 000 to 600 000 units or 10 000 IU/kg of vitamin D_3_ (5,10). Our clinical experience has shown us that there is poor patient compliance to long-term low-dose vitamin D applications, which may be insufficient to achieve the desired level of vitamin D. We use a 25-OH-D level below 20 ng/mL as a threshold value for pharmacological treatment in our clinic. We prefer stoss therapy rather than long-term low-dose vitamin D administration because of better patient compliance. Indeed, in a previous study, half of 42 infants and children aged between 5 months and 3 years with a 25-OH-D level of <20 ng/mL had been treated with stoss therapy (150 000 IU, single dose, oral) and the remaining were given low-dose long-term vitamin D_3_ therapy (2000 IU/day for 6 weeks) and a better vitamin D level was provided in the stoss therapy group without any side effects observed (11).

The clinical signs of vitamin D deficiency and insufficiency can be variable; the patient may be asymptomatic, have nonspecific clinical findings, or obvious rickets signs (4). Studies have shown that the risk of fracture is increased in children with low levels of vitamin D and that these children are also sensitive to respiratory tract infections (12,13). In the present study, none of the patients had a history of clinically significant fracture. According to the parents’ self-reports, 12.5% of the patients had experienced frequent respiratory tract infections. Additionally, there were some nonspecific clinical symptoms in the majority of the patients that could not be explained by any illness at admission, and these symptoms showed a decrease after vitamin D treatment (Figure 2). However, no relationship was found between these symptoms and the levels of vitamin D. In a previous study, Voloc et al (14) have reported poor correlation between clinical features and serum 25-OH-D levels.

All available evidence suggests that children and adults should maintain a blood level of 25-OH-D above 20 ng/mL to prevent rickets and osteomalacia. However, to maximize the effects of vitamin D on calcium level, bone, and muscle metabolism, it has been reported that the 25-OH-D blood levels should be above 30 ng/mL. Numerous epidemiological studies suggest that a 25-OH-D serum level above 30 ng/mL may have additional health benefits such as reducing the risk of common cancers, autoimmune diseases, type 2 diabetes, cardiovascular disease, and infectious diseases (8,15). In the present study, both treatment protocols were effective in providing a sufficient level of vitamin D, which remained higher in the 10 000 IU/kg treatment protocol group compared to the other protocol at the second week. However, the levels of vitamin D were decreased below the optimal level (≥30 ng/mL) at week 12 of the treatment in the majority of patients in both groups. Similar to our findings, in a randomized controlled study, half of the patients with rickets (mean age 12 months) received a 300 000 IU dose (Group 1) and the other half received a 600 000 IU dose (Group 2) of oral vitamin D_3_ therapy. The levels of 25-OH-D studied 12 weeks after initiation of the treatment were found to be below 20 ng/ mL in 62.5% of the patients in Group 1 and 64.3% of the patients in Group 2. In that study, the authors reported that both regimens failed to optimize the level of vitamin D for more than 3 months, but that no side effects were observed with either regimen (16). Although studies on this topic are limited, both our results and the data provided by Mittal et al (16) suggest that stoss therapy protocols should be repeated once every three months, especially in patients at risk for vitamin D deficiency with a poor compliance to vitamin D supplementation.

There is a lack of consensus on the dose of vitamin D_3_ in stoss therapy because of conflicting results from numerous studies. Stoss therapy can lead to side effects such as hypercalcemia, hypercalciuria, and nephrocalcinosis if vitamin D deficiency is not documented before therapy. Studies that compared low- and high-dose stoss therapy in nutritional rickets showed that 150 000 and 300 000 IU of vitamin D were adequate as treatment, but a 600 000 IU dose of vitamin D carried a risk of hypercalcemia (17,18). However, there are also studies reporting the use of a dose of 600 000 IU in the treatment of rickets due to vitamin D deficiency without any side effects (19,20). Lubani et al (19) showed that intramuscular vitamin D in a dose of 600 000 IU was safe and effective. In Shah and Finberg’s study (20), a single dose (600 000 IU) of vitamin D was given to 42 vitamin D-deficient children aged between 5 and 19 months; biochemical improvement was detected in 4-7 days and radiological improvement was detected in 10-14 days. Side effects such as hypercalcemia or hypercalciuria were not observed. In a recent meta-analysis, no risk was found for hypercalcemia or hypercalciuria in stoss therapy with a single oral dose below 400 000 IU, whereas doses above 400 000 IU created a risk for hypercalcemia (5). In the present study, none of the patients in either group developed hypercalcemia, hypercalciuria, vitamin D intoxication, or nephrolithiasis.

There are some limitations of this study that need to be acknowledged. Because of its retrospective design, we do not know about the calcium and vitamin D content of the patients’ diets and we have no evidence regarding the cause and effect relationship between the clinical symptoms and vitamin D deficiency at admission.

Unlike other studies in literature, this study has been performed in subjects who had no obvious clinical or radiological signs of rickets. This study shows that stoss therapy could be used in these groups of patients safely without any serious side effects.

In conclusion, our study demonstrated that the two stoss therapy protocols, namely 10 000 IU/kg and 300 000 IU doses, used in vitamin D insufficiency or deficiency patients who showed no marked signs of rickets seem to be similar in terms of efficacy and side effects. The study also showed that an optimal serum level of 25-OH-D cannot be maintained for more than three months and the treatment dose should be repeated at the 12^th^ week. We believe that these stoss therapy protocols can be used safely, especially in cases with poor patient compliance to vitamin D supplementation and in cases in which the optimal level of vitamin D cannot be achieved despite supplementation complemented by adequate nutritional approaches and encouragement of sunbathing.

## Figures and Tables

**Table 1:**
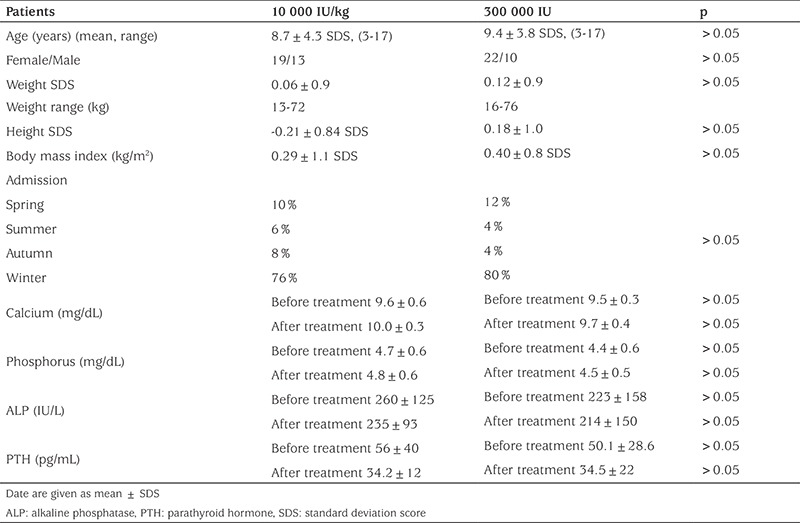
Clinical and laboratory characteristics of the patients before and two weeks after treatment

## References

[1] Carmeliet G, Dermauw V, Bouillon R (2015). Vitamin D signaling in calcium and bone homeostasis: a delicate balance. Best Pract Res Clin Endocrinol Metab.

[2] Spedding S, Vanlint S, Morris H, Scragg R (2013). Does vitamin D sufficiency equate to a single serum 25-hydroxyvitamin D level or are different levels required for non-skeletal diseases?. Nutrients.

[3] Manson JE, Bassuk SS (2015). Vitamin D research and clinical practice: at a crossroads. JAMA.

[4] Ozkan B (2010). Nutritional rickets. J Clin Res Pediatr Endocrinol.

[5] McNally JD, Iliriani K, Pojsupap S, Sampson M, O’Hearn K, McIntyre L, Fergusson D, Menon K (2015). Rapid normalization of vitamin D levels: a meta-analysis. Pediatrics.

[6] Holick MF, Binkley NC, Bischoff-Ferrari HA, Gordon CM, Hanley DA, Heaney RP, Murad MH, Weaver CM (2012). Guidelines for preventing and treating vitamin D deficiency and insufficiency revisited. J Clin Endocrinol Metab.

[7] Aloia JF (2011). Clinical Review: The 2011 report on dietary reference intake for vitamin D: where do we go from here?. J Clin Endocrinol Metab.

[8] Holick MF, Binkley NC, Bischoff-Ferrari HA, Gordon CM, Hanley DA, Heaney RP, Murad MH, Weaver CM, Endocrine Society (2011). Evaluation, treatment, and prevention of vitamin D deficiency: an Endocrine Society clinical practice guideline. J Clin Endocrinol Metab.

[9] Abrams SA, Committee on Nutrition (2013). Calcium and vitamin D requirements of enterally fed preterm infants. Pediatrics.

[10] Pettifor JM (2005). Rickets and vitamin D deficiency in children and adolescents. Endocrinol Metab Clin North Am.

[11] Emel T, Doğan DA, Erdem G, Faruk O (2012). Therapy strategies in vitamin D deﬁciency with or without rickets: efﬁciency of low-dose stoss therapy. J Pediatr Endocrinol Metab.

[12] Esposito S, Lelii M (2015). Vitamin D and respiratory tract infections in childhood. BMC Infect Dis.

[13] Gatti D, El Ghoch M, Viapiana O, Ruocco A, Chignola E, Rossini M, Giollo A, Idolazzi L, Adami S, Dalle Grave R (2015). Strong relationship between vitamin D status and bone mineral density in anorexia nervosa. Bone.

[14] Voloc A, Esterle L, Nguyen TM, Debray O, Colofitchi A, Jehan F, Garabedian M (2010). High prevalence of genu varum/valgum in European children with low vitamin D status and insufficient dairy products/calcium intakes. Eur J Endocrinol.

[15] IOM (Institute of Medicine) (2011). Dietary reference intakes for calcium and vitamin D. Washington DC: The National Academies Press.

[16] Mittal H, Rai S, Shah D, Madhu SV, Mehrotra G, Malhotra RK, Gupta P (2014). 300,000 IU or 600,000 IU of oral vitamin D3 for treatment of nutritional rickets: a randomized controlled trial. Indian Pediatr.

[17] Özkan B, Büyükavcı M, Energin M, Dirican ME (2000). Comparison of different treatment modalities (300,000 U oral, 300,000 U IM, 600,000 U oral vitamin D) in nutritional rickets. Çocuk Sağlığı ve Hastalıkları Dergisi.

[18] Cesur Y, Caksen H, Gündem A, Kirimi A, Odabaş D (2003). Comparison of low and high dose of vitamin D treatment in nutritional vitamin D deficiency rickets. J Pediatr Endocrinol Metab.

[19] Lubani MM, al-Shab TS, al-Saleh QA, Sharda DC, Quattawi SA, Ahmed SA, Moussa MA, Reavey PC (1989). Vitamin D-deficiency rickets in Kuwait: the prevalence of a preventable disease. Ann Trop Paediatr.

[20] Shah BR, Finberg L (1994). Single-day therapy for nutritional vitamin D-deficiency rickets: a preferred method. J Pediatr.

